# Hydrodynamic Effects on Biofilm Development and Recombinant Protein Expression

**DOI:** 10.3390/microorganisms10050931

**Published:** 2022-04-29

**Authors:** Alexandra Soares, Luciana C. Gomes, Gabriel A. Monteiro, Filipe J. Mergulhão

**Affiliations:** 1 LEPABE—Laboratory for Process Engineering, Environment, Biotechnology and Energy, Faculty of Engineering, University of Porto, Rua Dr. Roberto Frias, 4200-465 Porto, Portugal; up201711436@edu.fe.up.pt (A.S.); luciana.gomes@fe.up.pt (L.C.G.); 2 ALiCE—Associate Laboratory in Chemical Engineering, Faculty of Engineering, University of Porto, Rua Dr. Roberto Frias, 4200-465 Porto, Portugal; 3 IBB—Institute for Bioengineering and Biosciences, Department of Bioengineering, Instituto Superior Técnico, Universidade de Lisboa, 1049-001 Lisboa, Portugal; gabmonteiro@tecnico.ulisboa.pt

**Keywords:** biofilm, flow cell system, *Escherichia coli*, green fluorescent protein, hydrodynamic conditions, plasmid copy number

## Abstract

Hydrodynamics play an important role in the rate of cell attachment and nutrient and oxygen transfer, which can affect biofilm development and the level of recombinant protein production. In the present study, the effects of different flow conditions on the development of *Escherichia coli* biofilms and the expression of a model recombinant protein (enhanced green fluorescent protein, eGFP) were examined. Planktonic and biofilm cells were grown at two different flow rates in a recirculating flow cell system for 7 days: 255 and 128 L h^−1^ (corresponding to a Reynolds number of 4600 and 2300, respectively). The fluorometric analysis showed that the specific eGFP production was higher in biofilms than in planktonic cells under both hydrodynamic conditions (3-fold higher for 255 L h^−1^ and 2-fold higher for 128 L h^−1^). In the biofilm cells, the percentage of eGFP-expressing cells was on average 52% higher at a flow rate of 255 L h^−1^. Furthermore, a higher plasmid copy number (PCN) was obtained for the highest flow rate for both planktonic (244 PCN/cell versus 118 PCN/cell) and biofilm cells (43 PCN/cell versus 29 PCN/cell). The results suggested that higher flow velocities promoted eGFP expression in *E. coli* biofilms.

## 1. Introduction

Recombinant DNA methodology is very important for biotechnological industries because by using this technology, it is possible to produce proteins at much higher levels than are found naturally [1,2]. Although most heterologous proteins are produced on an industrial scale in suspended cells, it is known that the high-level production of heterologous proteins using planktonic cells has an associated metabolic burden on the host cell that can affect plasmid stability and protein yield [3]. Bacterial biofilms have recently received increased attention in terms of the production of added value compounds since they present many advantages over their free and planktonic counterparts, such as a higher cell density, protection against hostile conditions, higher operation stability [4,5], and greater plasmid stability [6,7]. Biofilms are communities of surface-attached microorganisms that are encased in a self-produced matrix of extracellular polymeric substances (EPS) [8]. Bacterial biofilms can contain multiple cell layers and their thickness can vary from a few to many μm [8]. The biological organization of biofilms can promote different rates of growth and metabolic activity within the biofilm cells, which can be a consequence of different diffusion rates of oxygen and nutrients inside the biofilms [9]. Microbial adhesion and the subsequent biofilm development can be affected by several factors, such as nutrient levels, surface properties, and hydrodynamic conditions [10,11,12,13].

Hydrodynamics is one of the most important factors that affects biofilm development since it determines the shear stress on the surfaces on which the biofilms form and the mass transfer of nutrients, oxygen, and bacteria from the bulk medium to the biofilm [14,15,16]. A higher flow velocity enables a higher external mass transfer, which means that more nutrients and oxygen are brought to the surface of the biofilm [15]. Hydrodynamic conditions also influence biofilm formation and structure due to the shear forces, which can modulate microbial cell adhesion to and detachment from a given surface [17]. A higher shear stress usually results in thinner, denser, and stronger biofilm, while lower shear stress conditions result in biofilms with thick multilayer structures [8,18]. Although a higher flow velocity increases the supply of nutrients and oxygen, the biofilm density can reduce the diffusion or penetration of the substrate into the inner layers of the biofilm [19].

The successful production of recombinant proteins in bacterial cells depends on sufficient nutrients and oxygen supply. Although biofilm reactors have been shown to have several advantages over planktonic cultures for recombinant protein production, the diffusion of substrates into the sessile cells is still a significant limitation [5,20]. It has also been shown that nutrients are mostly consumed by the outer cell layers before reaching the inner layers of the biofilm [19,21]. Recently, our research group demonstrated that the eGFP expression in *E. coli* biofilms is highly heterogeneous, with the cells that express the protein being located mostly on the top of the biofilm [22]. This is probably due to limitations in the mass transfer of oxygen and nutrients from the bulk liquid to the biofilm. Nevertheless, Huang et al. [23] showed that diffusion limitations can have a beneficial effect on plasmid stability since they can lower the cell growth rates inside the biofilm. Recombinant protein production in biofilm cells has been mostly performed in static conditions or using low laminar flow rates (Reynolds numbers (Re) of 20 and 32) [6,23,24,25]. Despite the lack of information on recombinant protein production in biofilms, it has been reported that agitation rate plays an important role in nutrient and oxygen diffusion within immobilized cells systems [26]. By using immobilized cells in sodium alginate for the production of recombinant aspartase, Singh and Yadav [27] demonstrated that a higher production rate (1234 U/g wet cells) was obtained with an agitation rate of 250 rpm compared to the lower agitation of 130 rpm (534 U/g wet cells). However, a high agitation rate (270 rpm) decreased the production to 931 U/g wet cells. Nevertheless, Man et al. [28] observed that an increased agitation rate negatively affected plasmid stability in the immobilized cells. However, the higher plasmid stability that was obtained at lower agitation rates was not associated with the increased excretion of the cyclodextrin glucanotransferase enzyme [28].

The aim of this work was to investigate the effects of flow rate variation on the development of *E. coli* biofilms and eGFP expression. For that, two flow conditions were tested: 255 and 128 L h^−1^ (corresponding to Re of 4600 and 2300, respectively). The flow cell system that was used in this study has been extensively characterized in terms of its hydrodynamics [15,16,29,30]. Turbulent conditions (Re of 4600) have already been tested in previous works using the same flow cell system for eGFP production [22,31], but laminar (Re < 2300) and transient conditions (2300 < Re < 4000) could also be achieved with this system. The variations in this operational parameter are discussed here for the first time.

## 2. Materials and Methods

### 2.1. Bacterial Strain

The *E. coli* JM109(DE3) (Promega, Madison, WI, USA) was transformed by heat shock with the plasmid pFM23 (constructed from pET28A; Novagen, Madison, WI, USA), which contained the eGFP gene [32]. The transformants were selected on an agar medium that was supplemented with 20 μg mL^−1^ of kanamycin (Eurobio, Orsay, France).

### 2.2. Flow Cell System and Experimental Conditions 

A flow cell system (Figure S1 of Supplementary Materials) composed of a vertical semicircular flow cell with removable coupons on which biofilms were formed and a recirculating tank (planktonic cells) was used, as described by Gomes et al. [22]. *E. coli* cells were grown by recirculating the bacterial suspension at 30 °C for 7 days at two different flow rates: 255 L h^−1^ (Re of 4600) and 128 L h^−1^ (Re of 2300). The Reynolds number was defined as (Equation (1)):

(1) $$Re=\frac{\rho UD_{h}}{\mu }$$

where ρ is the density of water at 30 °C (kg m^−3^), U is the linear flow velocity (m s^−1^), $D_{h}$ is the hydraulic equivalent diameter of the semicircular flow cell ($D_{h}\;$= πD/(2+π) = 1.83 cm) of diameter D (m), and μ is the dynamic viscosity of water at 30 °C (kg (m^−3^ s^−1^)).

The average wall shear stress ($\tau_{w}$) values for the two tested flow conditions (Re of 4600 and 2300) were estimated using the dimensionless Darcy friction factor (f), which was obtained from the work of Teodósio et al. [16], and applying Equation (2):

(2) $$f=\frac{4\tau_{w}}{\rho U^{2}/2}$$

where ρ is the density of water at 30 °C (kg m^−3^) and U is the linear flow velocity (m s^−1^). A Re of 4600 and 2300 in the flow cell system corresponded to $\tau_{w}$ values of approximately 0.3 and 0.1 Pa, respectively.

The recirculating tank was aerated using an air pump (flow rate of 108 L h^−1^) and continuously fed with Terrific Broth (TB) medium, which was supplemented with 20 µg mL^−1^ of kanamycin, at a flow rate of 0.025 L h^−1^ [22]. In a recent screening test, our research group found that the eGFP production was higher using the TB medium compared to Lysogenic Broth (LB) (data not shown). The TB medium is composed of 12 g L^−1^ tryptone, 24 g L^−1^ yeast extract, 2.31 g L^−1^ KH_2_PO_4_, and 12.54 g L^−1^ K_2_HPO_4_. All compounds were purchased from Merck, Algés, Portugal.

### 2.3. Biofilm and Planktonic Monitoring

For the biofilm sampling, the system was stopped every day for coupon removal and was then carefully restarted at the same flow rate [22]. The biofilm wet weight and thickness were firstly determined by subtracting the coupon weight on the sampling day from the weight prior to the start of the experiment and by using a digital micrometer, respectively [29]. Then, the biofilm was resuspended in 25 mL of 8.5 g L^−1^ NaCl solution for total, viable, and eGFP-expressing cells quantification and also for eGFP production and plasmid analysis. The biofilm total and viable cells were determined using the Live/Dead^®^ BacLight^TM^ bacterial viability kit (Invitrogen Life Technologies, Alfagene, Carcavelos, Portugal), as fully described by Gomes et al. [22]. Briefly, each biofilm cell suspension was filtered through a Nucleopore Track-Etch Membrane of black polycarbonate (pore size 0.22 µm; Whatman Ltd., Banbury, UK), stained for 7 min in the dark, and then observed using an epifluorescence microscope (Leica DM LB2; Leica Microsystems Ltd., Heerbrugg, Switzerland) that was connected to a Leica DFC300 FX camera. ImageJ v1.48 software (National Institutes of Health, Bethesda, MD, USA) was used to quantify the number of total and viable cells from the counts of a minimum of 15 fields of view per sample and the final values were expressed as log cells cm^−2^.

For planktonic cells, a daily 10 mL sample was taken from the recirculating tank to assess the total and viable cell numbers and to perform eGFP and plasmid analysis. The total and viable cell numbers were assessed using the same methodology as for the biofilm cells and the final values were presented as log cells mL^−1^.

#### 2.3.1. Assessment of eGFP-Expressing Cells and Specific Protein Concentrations

The eGFP-expressing cells were observed and counted using epifluorescence microscopy with a Leica DM LB2 epifluorescence microscope, as described by Gomes et al. [22]. The final values were presented as log cells mL^−1^ and log cells cm^−2^ for planktonic and biofilm samples, respectively. 

The specific eGFP production was analyzed using the fluorometric method that was described by Mergulhão and Monteiro [32]. A microplate reader (SpectraMax M2E, Molecular Devices, Inc., Wokingham, UK) was used to measure the eGFP fluorescence using an excitation filter of 488 nm and an emission filter of 507 nm. The eGFP concentration was then calculated using a standard curve that was obtained from purified eGFP (ranging from 0 to 0.31 µg µL^−1^), and presented as specific eGFP production (fg cell^−1^).

#### 2.3.2. Plasmid Extraction and Quantification

The plasmid DNA was extracted using alkaline lysis and quantified using the analytical high-performance liquid chromatography (HPLC) method based on hydrophobic interaction chromatography (HIC), as fully described by Soares et al. [7]. A 4.6/100 mm HIC source of 15 PHE PE columns (Amersham Bioscience, Uppsala, Sweden) that was connected to an HPLC system (Shimadzu, Nexera-i LC-2040C 3D, Duisburg, Germany) was used. The plasmid concentration was calculated using calibration curves that were constructed from the serial dilutions of the plasmid DNA standards (ranging from 0.04 to 20 µg mL^−1^). Then, the corresponding plasmid copy number (PCN) was calculated using Equation (3) [33]:

(3) $$\mathrm{PCN}=\frac{6.02\times 10^{23}\;\left({\mathrm{copy}\;\mathrm{mol}}^{-1}\right)\times \mathrm{DNA}\;\mathrm{amount}\;\left(g\right)}{\mathrm{DNA}\;\mathrm{length}\;\left(\mathrm{bp}\right)\times 660\;\left({g\;\mathrm{mol}}^{-1}{\;\mathrm{bp}}^{-1}\right)}$$

where the size of plasmid pFM23 was 6053 bp.

### 2.4. Statistical Analysis 

**Figure 1:**
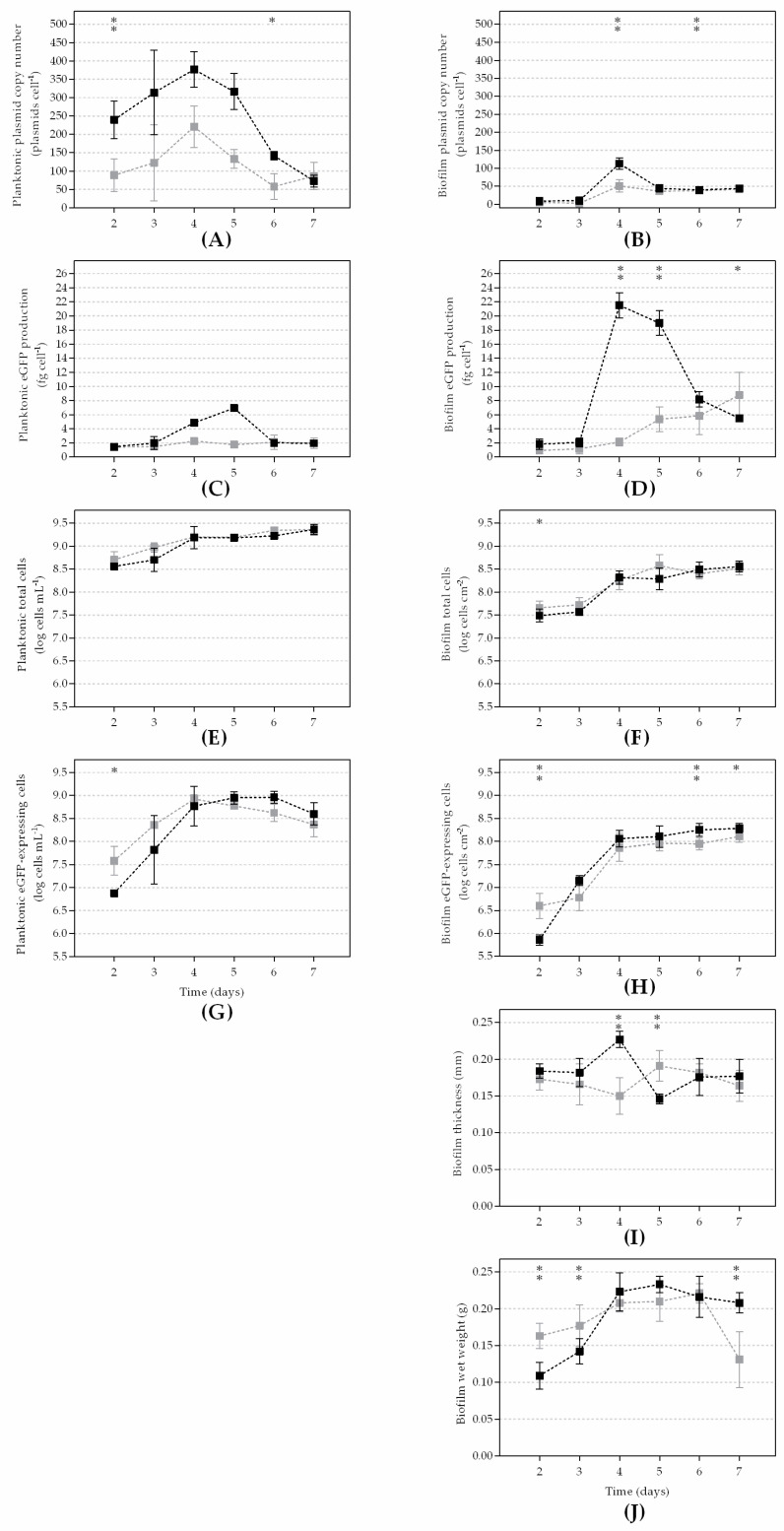
Planktonic and biofilm parameters at a flow rate of 255 L h^−1^ (Re of 4600; dark squares) and 128 L h^−1^ (Re of 2300; gray squares): (**A**) planktonic plasmid copy number; (**B**) biofilm plasmid copy number; (**C**) planktonic eGFP production; (**D**) biofilm eGFP production; (**E**) planktonic total cells; (**F**) biofilm total cells; (**G**) planktonic eGFP-expressing cells; (**H**) biofilm eGFP-expressing cells; (**I**) biofilm thickness; and (**J**) biofilm wet weight. The means ± standard deviations (SDs) for at least three independent experiments are illustrated. The statistical analysis corresponding to each time point is also represented with an asterisk for a confidence level of greater than 90% (*p* < 0.1) and with a double asterisk for a confidence level of greater than 95% (*p* < 0.05).

The results that are presented in Figure 1 and Figure S1 (Supplementary Materials) were the averages of at least three independent experiments for each flow rate condition (255 and 128 L h^−1^). All reported data are presented as the mean ± standard deviation (SD) from the three replicates of each independent experiment. 

A paired *t*-test analysis using GraphPad Prism 6.01 software (San Diego, CA, USA) was performed based on a confidence level of 90% (differences are reported as significant for *p* values < 0.1) and a confidence level of 95% (differences are reported as significant for *p* values < 0.05).

## 3. Results

In this study, the effects of hydrodynamics on eGFP production and plasmid stability in *E. coli* planktonic and biofilm cells were assessed by testing two different flow rates: 255 L h^−1^ (Re of 4600) and 128 L h^−1^ (Re of 2300).

The number of plasmids per cell (PCN/cell; Figure 1A,B) was higher at the highest flow rate for both planktonic and biofilm cells (on average 2- and 1.5-fold higher, respectively). In planktonic cells (Figure 1A), PCN increased until day 4, on which it reached a maximum value of 377 PCN/cell for the highest flow rate and 221 PCN/cell for the lowest flow rate that was tested. After that PCN peak, a continuous decrease was observed until the end of the experiment. The same initial behavior was observed in the biofilm cells (Figure 1B), with a maximum value of 113 PCN/cell for the highest flow rate and 51 PCN/cell for the lowest flow rate on day 4. From day 4 onwards, PCN decreased and reached a steady-state of 42 PCN/cell for the highest Re and 38 PCN/cell for the lowest Re. 

Although higher PCN values were always achieved at the highest flow rate, the plasmid maintenance in both planktonic and biofilm cells seemed to be favored at a lower Re. In fact, for the planktonic cells that were grown at 255 L h^−1^, a strong reduction was observed between days 4 and 7 (around 81%), while at a flow rate of 128 L h^−1^ plasmid loss was around 61%. Regarding the biofilm cells, the percentage of plasmid lost at 255 L h^−1^ was 63%, whereas at 128 L h^−1^ this reduction was approximately 25%. 

The fluorometric analysis of the biofilm cells (Figure 1D) showed that the cells grown at the highest Re expressed more eGFP and reached the highest production value of 21.5 fg cell^−1^ on day 4. After day 4, the production decreased until the end of the experiment. Additionally, the eGFP production rate that was obtained at the lowest flow rate remained almost constant until day 4 and after that, it started to increase until the end of the experiment and reached a maximum of 8.8 fg cell^−1^. In the planktonic state (Figure 1C), the production of eGFP was also higher at the highest flow rate and increased until day 5 (7.0 fg cell^−1^), whereas at the lowest flow rate the eGFP levels remained practically constant over the whole experiment (around 1.8 fg cell^−1^). Furthermore, the protein production rate in the biofilm cells was higher compared to the planktonic cells under both hydrodynamic conditions (3-fold higher for 255 L h^−1^ and 2-fold higher for 128 L h^−1^).

The planktonic total cells (Figure 1E) had similar profiles for both flow rates (*p* > 0.1) and throughout the whole experiment. The quantification of the biofilm total cells (Figure 1F) also showed few differences between the hydrodynamic conditions. An initial increase was observed until day 4 and then, the number of cells stabilized from day 4 onwards. Concerning the planktonic eGFP-expressing cells (Figure 1G), both flow conditions reached similar cell amounts (8.6 × 10^8^ cells mL^−1^), although this value was reached sooner for the flow rate of 128 L h^−1^ (day 4 versus day 5 for 255 L h^−1^). From that day onwards, the number of cells that expressed eGFP decreased until the end of the experiment. By comparing the number of planktonic cells that were expressing eGFP to the total cell number, the results showed that on average 31% and 26% of the cells expressed eGFP at 255 L h^−1^ and 128 L h^−1^, respectively (data not shown). The number of biofilm cells that were expressing eGFP (Figure 1H) strongly increased between days 2 and 4 and then remained practically constant until the end of the experiment, regardless of the hydrodynamic condition being tested. The number of cells that were expressing eGFP in comparison to the total cell number was on overage 53% higher for the highest flow rate. Under this flow condition, a strong increase was observed until day 5, when 66% of the cells were found to express the protein. Furthermore, for the flow rate of 255 L h^−1^, the number of biofilm cells that expressed eGFP was on overage 52% higher than the planktonic counterparts.

The biofilm thickness (Figure 1I) remained practically constant throughout the experiment for both flow conditions (an average of 176 µm), with a slight increase on days 4 and 5 for 255 L h^−1^ and 128 L h^−1^, respectively. The biofilm wet weight (Figure 1J) was similar between flow rates but had slightly higher values for 255 L h^−1^. An initial increase was observed until day 4, which then stabilized from that day until the end of the experiment for 255 L h^−1^, while a decrease was observed on the last day of the experiment for the flow rate of 128 L h^−1^. 

## 4. Discussion

The study of the parameters that influence both biofilm formation and heterologous protein production is a valuable approach for reaching the highest specific recombinant protein expression in *E. coli* biofilms. Previous experiments using the same flow cell system have shown that higher flow velocities increase the external mass transfer [14,15] but produce thinner and more compact biofilms (compared to laminar conditions) [34] in which the internal mass transfer can be limited [22]. As well as a turbulent regime (flow rate of 255 L h^−1^ and Re of 4600), transient conditions (flow rate of 128 L h^−1^ and Re of 2300) were also tested in the present work.

Doubling the flow rate (from 128 to 255 L h^−1^) seemed to have no impact on biofilm formation. This result was not expected since it is generally accepted that biofilms that are formed under turbulent conditions are thinner, denser, and stronger than those developed under lower shear stress [15,29]. This could be related to the presence of an antibiotic resistance gene (kanamycin, KmR) in the plasmid. It is known that these genes are the major genes responsible for increasing the metabolic burden on the host cell [35]. Since stress conditions can favor biofilm formation [36] and, additionally, the presence of a considerable number of eGFP-expressing cells can increase the stress, it is possible that the similar biofilm growth behavior under the different flow conditions could have been due to these factors [31,36]. 

The single-cell analysis showed an increase in the number of eGFP-expressing biofilm cells between days 2 and 5, which represented on average 58% and 37% of the total population for 255 and 128 L h^−1^, respectively, from this moment until the end of the experiment. It should be noted that, contrary to other studies, the number of eGFP-expressing cells did not correlate with the number of viable cells (Figure S2 of Supplementary Materials). In fact, the number of eGFP-expressing cells was found to be higher than viable cells under both flow conditions. This could be explained by the fact that dead cells completely lose eGFP while dying cells gradually leak eGFP from increasingly permeable membranes [37] once a decrease in viability has been observed. The decrease in biofilm viability could be related to the increase in protein production, which imposes a metabolic burden on the host [38,39]. It is well documented that recombinant protein production affects host metabolism due to the energy and metabolites that are needed for the replication of plasmid DNA and the synthesis of foreign protein [3,40]. Some of the metabolic changes that can be induced during recombinant protein production are an increase in protease activity and a decrease in growth rate and cell viability [38,39]. 

The specific eGFP production was also higher for both planktonic and biofilm cells that were grown at the 255 L h^−1^ flow rate. In suspended cells, several studies have reported that a high agitation rate increases metabolite production in recombinant microorganisms [41,42,43]. This suggests that a high shear rate is generated under high flow velocities and results in an increase in oxygen and nutrient supply. Some studies of recombinant protein production in immobilized bacterial cells have also demonstrated the influence of agitation rate on oxygen and nutrient diffusion within the cells and the consequent effects on heterologous protein production [27,28]. Talabordan and Yang [44] reported that the production of a glucoamylase–green fluorescent fusion protein on a fibrous-bed bioreactor increased when the agitation rate increased to 400 rpm and that a further increase to 600 rpm resulted in the detachment and breakage of fungal mycelia and a consequent increase in protein secretion. Similar to bacterial biofilms, filamentous microorganisms comprise channels that allow fluid circulation when immobilized in reactors [45]. An increase in agitation rate promotes a better mass transfer within the cells [45]. Biofilms are known to be highly heterogeneous and contain cells in different physiological states, which influence gene expression in different zones within the biofilm [19,46,47]. In a previous work by our group [22], it was demonstrated that the eGFP-expressing cells in a 3-day *E. coli* biofilm were mostly found at the top of the biofilm. The lower eGFP expression that was obtained at the lowest tested flow rate in this study (128 L h^−1^) could be related to the absence of oxygen and fresh nutrients in the deeper regions of the biofilm. In agreement with this hypothesis, Araújo et al. [34] showed that mass transfer limitations in *Pseudomonas fluorescens* biofilms occured under lower velocity conditions (0.1 m s^−1^, which corresponded to a Reynolds number of 1000). Additionally, the higher eGFP production that was obtained at the higher flow rate was probably related to the high availability of oxygen and nutrients in the bulk fluid, since a direct consequence of increasing flow velocity is that the transport of nutrients and oxygen also increases [48]. 

Although the specific eGFP production in the biofilms that were grown at the lower flow rate remained almost constant until day 4, an increase was observed from that day onward. This phenomenon could be associated with the slight increase that was observed in PCN and the percentage of eGFP-expressing cells during the same period. It was expected that the higher PCN and the larger number of cells that were expressing the protein would result in higher eGFP levels. Furthermore, the increase in the number of eGFP-expressing cells on day 4 could be related to the simultaneous decrease in biofilm thickness, since the oxygen transfer that is needed for the maturation of eGFP is facilitated in thinner biofilms.

Higher specific eGFP production levels were obtained in biofilm cells, even though they presented a smaller number of plasmid copies compared to the planktonic cells. These results were in agreement with our previous work [7], in which eGFP production was shown to be higher in biofilm cells, even with 3-fold lower PCN levels. Thus, under the tested hydrodynamic conditions, gene dosage did not affect recombinant protein production. This was probably related to the metabolic burden that was imposed by plasmid replication, which can promote plasmid instability and, consequently, reduce the yield of heterologous protein in planktonic cells [3,49]. 

Biofilm environments have been shown to increase plasmid maintenance compared to chemostats [6,7,50]. In this work, a higher percentage of plasmid loss was observed in the planktonic state for both flow rates. However, a low flow rate seemed to be advantageous for plasmid retention: in biofilm cells, 25% of plasmid loss was observed at 128 L h^−1^ while at a higher flow rate, this percentage was 2.5-fold higher. The marked decline in PCN on day 5 for the biofilm cells that were grown at 255 L h^−1^ could be related to the higher eGFP production that was observed on days 4 and 5. It is known that higher levels of protein production increase the metabolic burden on the host cell, which can contribute to plasmid loss [3,51]. Huang et al. [26] reported that biofilm cells that are grown at low agitation rates are associated with high plasmid stability.

## 5. Conclusions

Despite the advantages that biofilm reactors present over suspended cells, limitations in the diffusion of oxygen and nutrients within the biofilm remain a major problem. This study demonstrated that a flow rate had an impact on recombinant protein production in *E. coli* biofilms. Although increasing the flow rate had no major impact on biofilm formation, higher eGFP production and PCN values were obtained at the higher flow rate. The results suggested that the higher hydrodynamics favored the recombinant protein production in *E. coli* biofilms. 

The results that were obtained in this work are essential for the definition of the operating conditions that are needed for heterologous protein production in biofilms systems in order to maximize protein production.

## Data Availability

The data presented in this study are available on request from the corresponding author. The data are not publicly available yet as some datasets are being used for additional publications.

## Supplementary Materials

The following supporting information can be downloaded at: https://www.mdpi.com/article/10.3390/microorganisms10050931/s1, Figure S1: (A) Schematic representation and (B) photograph of the flow cell system, Figure S2: Planktonic (A) and biofilm (B) viable cells at the 255 L h^−1^ flow rate (Re of 4600; dark square) and 128 L h^−1^ flow rate (Re of 2300; gray square). The means ± standard deviations (SDs) for at least three independent experiments are illustrated. The statistical analysis corresponding to each time point is also represented with a double asterisk for a confidence level of greater than 95% (*p* < 0.05).
