# Supplementary material for: Hydrodynamic Effects on Biofilm Development and Recombinant Protein Expression
Source: Microorganisms. 2022 Apr 29;10(5):931. doi: 10.3390/microorganisms10050931 (PMC9145004; doi:10.3390/microorganisms10050931)
Supplement: Supplementary file 1 [file microorganisms-10-00931-s001.zip › microorganisms-1685795-supplementary.pdf]

Supplementary Material

Flow cell system schematization and experimental procedure

The flow cell system is composed of a recirculating tank (planktonic cells), a vertical flow cell reactor (where biofilms are formed), and centrifuge pumps that allow the circulation of the bacterial suspension. The flow cell reactor is a semi-circular Perspex duct (3.0 cm in diameter and 1.09 m in length) with 21 apertures on its flat wall to fit removable Perspex pieces (coupons) to which polyvinyl chloride (PVC) slides (2 x 1 cm) were glued. The biofilms were developed on the upper faces of the PVC slides that were in contact with the bacterial suspension circulating through the system.

E. coli cells were grown by recirculating the bacterial suspension at 30 °C for 7 days at two different flow rates: 255 L h^{-1} (Re of 4600) and 128 L h^{-1} (Re of 2300). The recirculating tank was aerated using an air pump (flow rate of 108 L h^{-1}) and continuously fed with Terrific Broth (supplemented with 20 μg mL^{-1} kanamycin) at a flow rate of 0.025 L h^{-1}.

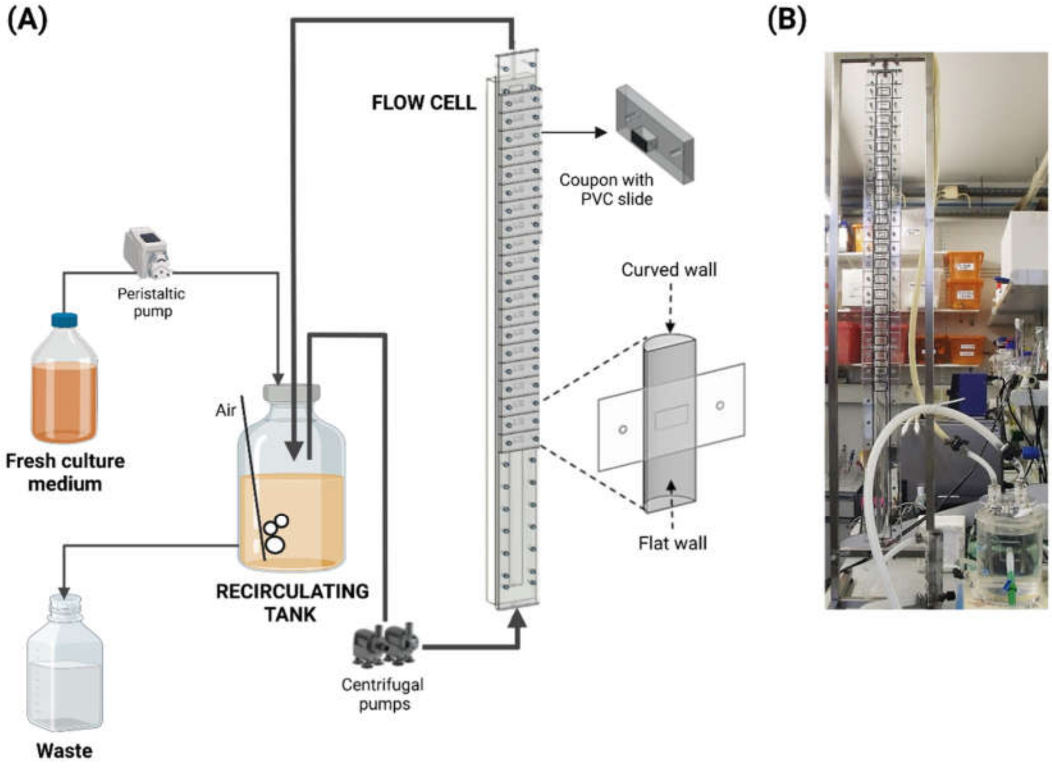

Figure S1. (A) Schematic representation and **(B)** photograph of the flow cell system.

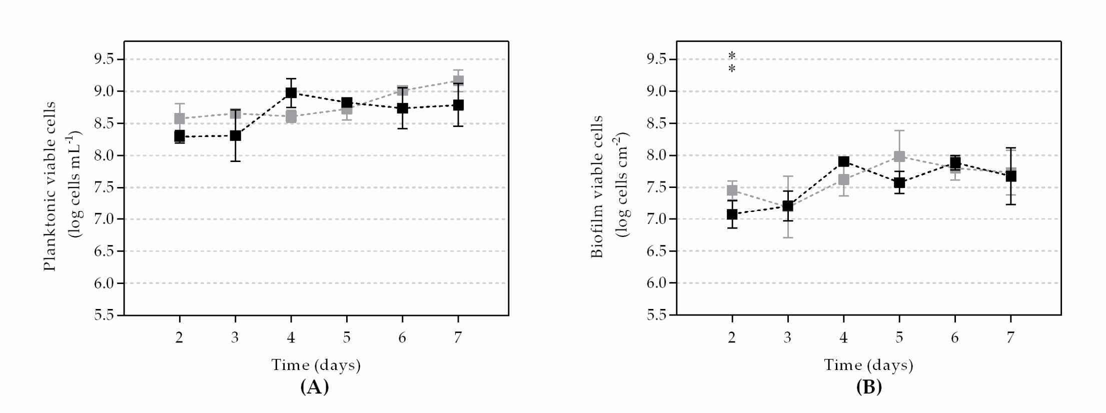

Figure S2. Planktonic **(A)** and biofilm **(B)** viable cells at a flow rate of 255 L h^{-1} (*Re* of 4600, dark squares) and 128 L h^{-1} (*Re* of 2300, grey squares). The means ± standard deviations (SDs) for at least three independent experiments are illustrated. Statistical analysis corresponding to each time point is also represented with an asterisk for a confidence level greater than 90% ($p < 0.1$) and with a double asterisk for a confidence level greater than 95% ($p < 0.05$).
